# Draft Genome Sequence of Saccharomyces cerevisiae Strain Pf-1, Isolated from Prunus mume

**DOI:** 10.1128/MRA.01169-19

**Published:** 2019-11-14

**Authors:** Shin Kanamasa, Daiki Yamaguchi, Chiyoko Machida, Tsukasa Fujimoto, Anna Takahashi, Masataka Murase, Shuichi Fukuyoshi, Akifumi Oda, Kenji Satou, Hiro Takahashi

**Affiliations:** a Graduate School of Bioscience and Biotechnology, Chubu University, Kasugai, Aichi, Japan; b College of Bioscience and Biotechnology, Chubu University, Kasugai, Aichi, Japan; c Graduate School of Horticulture, Chiba University, Matsudo, Chiba, Japan; d Faculty of Information Technologies and Control, Belarusian State University of Informatics and Radio Electronics, Minsk, Belarus; e Graduate School of Medical Sciences, Kanazawa University, Kanazawa, Ishikawa, Japan; f Institute of Medical, Pharmaceutical and Health Sciences, Kanazawa University, Kanazawa, Ishikawa, Japan; g Faculty of Pharmacy, Meijo University, Nagoya, Aichi, Japan; h Faculty of Biological Science and Technology, Institute of Science and Engineering, Kanazawa University, Kanazawa, Japan; i Fundamental Innovative Oncology Core Center, National Cancer Center, Tokyo, Japan; Broad Institute

## Abstract

Saccharomyces cerevisiae strain Pf-1 is a yeast isolated from Prunus mume; it potentially can be used to produce wine and traditional Japanese sake. Here, we report the draft genome sequence of this strain. The genomic information will provide a deeper understanding of the brewing characteristics of this strain.

## ANNOUNCEMENT

Wine is one of the oldest alcoholic beverages in the world. Seishu (refined sake), which is a traditional Japanese alcoholic beverage made from rice, is produced by advanced parallel double fermentation using the fungus Aspergillus oryzae and yeast Saccharomyces cerevisiae. With a few exceptions, alcoholic fermentation (ethanol production) by yeast is essential for the production of all alcoholic beverages (1). During the brewing process, each type of yeast species characteristically influences the flavor of the resulting beverage due to the production of various organic acids and incorporation of sugars and organic acids contained in the raw materials (2). Thus, each yeast strain is very important in the brewing process and ethanol production. This study aimed to develop wine and Seishu with unique characteristics using yeasts obtained from various plants. We previously described that S. cerevisiae strain Hm-1, isolated from the flower of cotton rosemallow (Hibiscus mutabilis), is suitable for brewing Seishu, and we analyzed the genome of this strain (3, 4). We also subsequently isolated the novel S. cerevisiae strain Pf-1 from the plum fruit (Prunus mume) and conducted brewing tests to prove that it can be used to produce sake with a unique flavor (5). We presently analyzed the genome sequence of strain Pf-1 to understand its brewing characteristics at the molecular level and to produce novel alcoholic beverages.

The genomic DNA of strain Pf-1 was isolated from a liquid-submerged culture grown in yeast extract-peptone-dextrose (YEPD) medium at 30°C. Using the NEBNext Ultra II FS DNA library prep kit for Illumina (New England BioLabs [NEB], MA, USA), a 500-ng DNA aliquot was fragmented using DNA fragmentase to generate DNA fragments (average, 600 bp) for library preparation (6, 7). TruSeq DNA library sequencing (paired-end 2 × 300-bp reads) generated 6,977,098 reads. Removal of the sequencing primers and trimming low-quality read regions from the obtained short reads were conducted using fastp version 0.20.0 (8) with the -c parameter. *De novo* assembly was conducted using the CLC Genomics Workbench version 12.0.2 (Qiagen, Valencia, CA, USA) with a word size of 60 and bubble size of 1,700. The parameters were manually and heuristically optimized to maximize the *N*_50_ contig length. Contigs shorter than 200 bp were discarded. The resulting genome assembly had a length of 12,480,617 bp, which was divided into 2,247 contigs. The *N*_50_ contig length was 102,829 bp, the G+C content was 38.5%, and the genome coverage was 162.1×. The coding regions of chromosomes were predicted using AUGUSTUS version 3.2.2 (9) and implemented in OmicsBox version 1.1.164 (Qiagen), with S. cerevisiae S288C as a gene model. The estimated number of genes in the draft genome was 5,732. The assembly contained 97.9% of the complete universal single-copy ortholog genes, as estimated by BUSCO version 3.1.0 (10). This genomic information can provide insights into the genetic basis of the brewing characteristics of this strain.

### Data availability.

The draft genome sequence for yeast strain Pf-1 has been deposited in GenBank/ENA/DDBJ under accession number BKZN00000000 (BKZN01000001 to BKZN01002247). The SRA/DRA/ERA accession number is DRA008974.
